# Recent advances in organic agriculture: innovations, challenges, and opportunities

**DOI:** 10.3389/fpls.2025.1681928

**Published:** 2026-01-09

**Authors:** Shweta Meshram, Haryati B. Mansor, Tika B. Adhikari

**Affiliations:** 1 Department of Plant Pathology, Lovely Professional University, Phagwara, India; 2 Organic Agriculture Program, Soil Science, Water and Fertilizer Research Centre Malaysian Agricultural Research and Development Institute (MARDI) Headquarters, Persiaran Malaysian Agricultural Research and Development Institute (MARDI)- Universiti Putra Malaysia (UPM), Serdang, Selangor, Malaysia; 3 Department of Entomology and Plant Pathology, North Carolina State University, Raleigh, NC, United States

**Keywords:** certification systems, disease management, policy frameworks, artificial intelligence, sustainable agriculture

## Abstract

Organic agriculture has become a more sustainable option compared to conventional agriculture, emphasizing biodiversity, healthy soils, and restrained pesticide applications. The purpose of this review is to integrate advances in cross-regional organic agriculture, with a special focus on how policy contexts, certification schemes, and technological advancements interact to influence adoption and sustainability levels. It highlights the developments, challenges, and sustainable outcomes of organic agriculture systems in four major regions of the world, including India, Europe, Malaysia, and the United States. Comparative analysis indicates that policy-based models, such as the EU’s Green Deal, which aims to have 25% of agricultural land under organic farming by 2030, have accelerated the adoption of organic agriculture. In contrast, U.S. systems, although yielding 10–18% less, have 22–35% higher profitability due to market incentives and USDA programs. It also seeks to contrast regional models of organic farming, providing a brief overview of policy regimes, certification systems, technological innovations, and disease management strategies in organic farming. In India, indigenous practices and Participatory Guarantee Systems (PGS) provide support to smallholder farmers. Europe stands in stark contrast to the overarching policy interventions outlined in the Green Deal. The United States focuses on market-led growth in the organic agriculture sector. Concurrently, Malaysia integrates government incentives, urban agriculture, and private-public partnerships, especially for highland regions like the Cameron Highlands, to encourage organic vegetable production. Despite the economic and environmental advantages of organic agriculture, it is facing regulatory complexity, the cost of certification, and yield gaps. Emerging evidence on artificial intelligence and precision technologies suggests enhanced efficiency in nutrient and pest management in organic systems. Together, these findings underscore the promise of organic agriculture, provided that future research targets low-cost biocontrols, climate-resilient varieties, and AI-based precision tools.

## Introduction

Organic agriculture (OA) has gained increasing attention worldwide as a sustainable alternative to conventional agriculture. Defined as an ecological production system that enhances biodiversity, restores soil health, and minimizes synthetic inputs, organic agriculture aligns closely with global sustainability goals (Gamage et al., 2023). By the end of 2023, the world’s organic farming land had been managed by 4.3 million organic farmers. At the same time, retail sales of organic food reached approximately USD 148 billion in 2023 (FiBL, 2023a; FiBL, 2023b; FiBL Statistics, 2023). As of 2023, organic farming covered 19.5 million hectares of agricultural land in Europe, of which 17.7 million hectares were within the European Union (FiBL Statistics, 2023).

Organic food sales in the United States have increased consistently at an average annual rate of approximately 7%, rising 3.4% in 2023, indicating further growth of the organic market nationally and internationally (the USDA, 2025b; USDA-ERS, 2023; The Organic Trade Association, 2023; Skorbiansky, 2025; Carlson et al., 2023). Similar trends are evident in Europe, Malaysia, and India, where consumer preferences for pesticide-free, environmentally friendly produce have driven the adoption of organic practices (Parikh, 2023; USDA, 2025a; 2025b).

**Figure 1:**
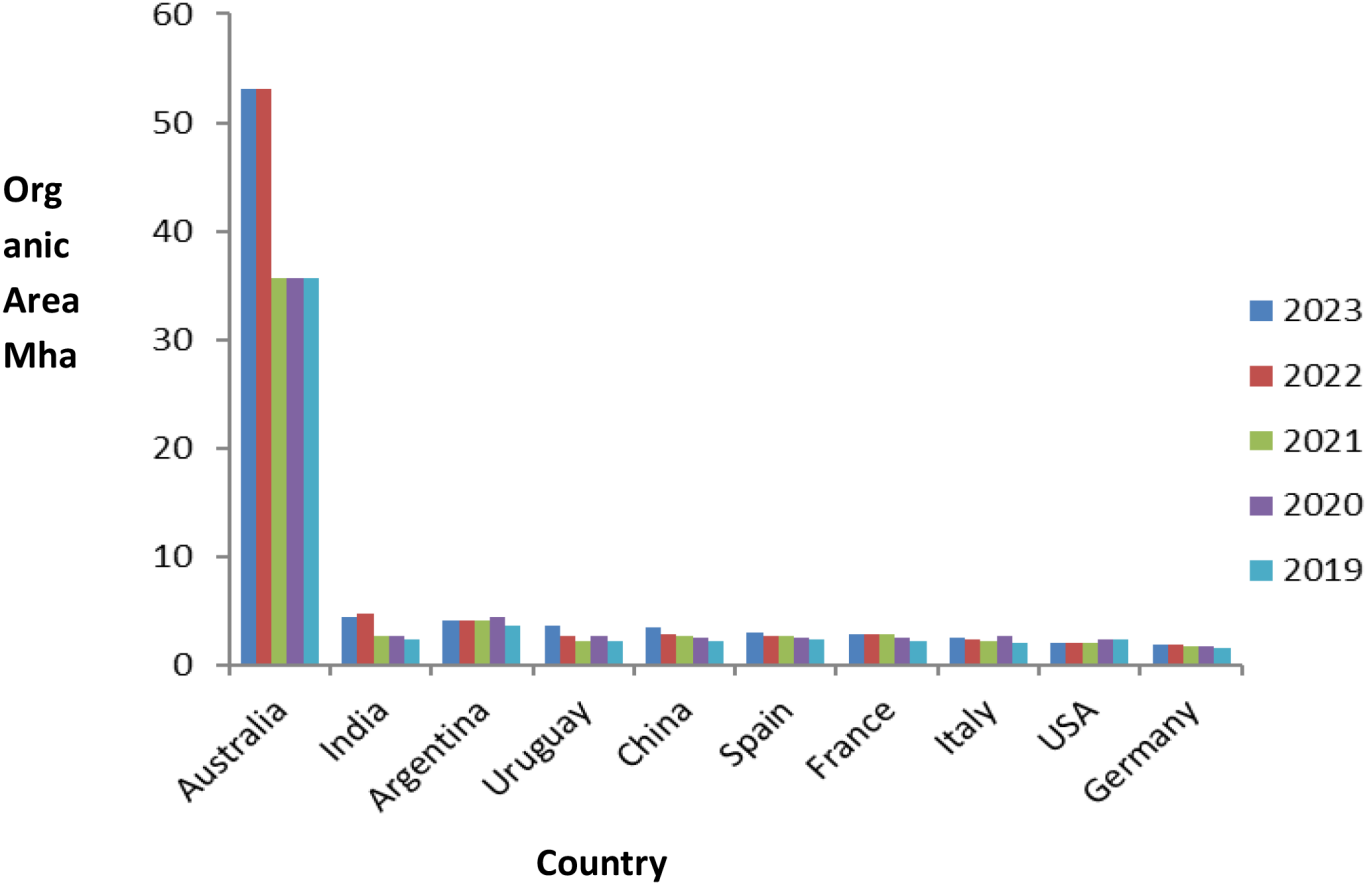
Bar chart displaying the organic area in million hectares (Mha) from 2019 to 2023 across various countries. Australia leads with the largest area, followed by India, Argentina, and others. Each country has bars for each year, with 2023 shown in blue, 2022 in red, 2021 in green, 2020 in purple, and 2019 in light blue. Data represents the total organic agricultural area (including cropland, pasture, and rangeland). Source: FiBL Statistics, 2023 and https://statistics.fibl.org/world/area-world.html.

In 2023, among the top 10 countries for certified organic agricultural areas, the USA ranks 9th globally, with over 2 million hectares dedicated to organic cultivation (**Figure 1**). High-value crops and federal policy initiatives primarily support this growth. In contrast, Malaysia is not among the top 10. Still, it has shown consistent growth due to initiatives such as the Malaysia Organic Scheme (myOrganic), urban farming efforts, and support for export-oriented high-value crops (Somasundram et al., 2016; Rahman et al., 2021). By 2023, Malaysia’s certified organic agricultural land area had reached approximately 1,386 hectares, according to FiBL statistics, and is projected to remain at this level by 2025.

The gaps in organic farming are complex and interconnected, spanning from financial limitations to societal attitudes. The economic cost of the shift to organic farming is the most daunting challenge for farmers. The practice of organic agriculture sometimes demands significant initial investments in organic inputs, including biofertilizers, organic seeds, and natural methods of pest control, which are often more costly than their traditional counterparts. Moreover, the period of transition, typically lasting two to three years, during which farmers must follow organic practices without the option of selling their products as organic, can be accompanied by smaller yields and lower revenue (Dou et al., 2025; Mahedi et al., 2025).

Organic agriculture focuses on enhancing soil fertility, conserving biodiversity, and promoting ecological resilience. Techniques such as crop rotation, composting, green manuring, and biological pest control not only improve soil structure and nutrient content but also reduce the risk of soil erosion and contamination (Padel, 2001; Padel et al., 2025; Panday et al., 2024). Research has demonstrated that organic soils contain higher levels of organic carbon, which enhances their ability to sequester carbon and mitigate the impacts of climate change (Niggli et al., 2017).

However, despite the rapid global expansion of organic agriculture, a research gap remains in cross-regional synthesis that links certification systems, technological innovations, and policy effectiveness.

### Review aim

This study aims to review the current state of organic farming, identify challenges, and compare regional models. It also provides an overview of policies, certification schemes, technological innovations, and strategies for managing plant diseases.

### Review objectives

1. To review the present OA production methods and technologies across various countries.
2. To assess the knowledge gaps and current challenges faced by OA systems.
3. To highlight future directions and innovative approaches that can enhance OA and improve disease management strategies.

This review offers a comparative overview of organic agriculture systems in India, Europe, Malaysia, and the United States. It provides a snapshot of organic agriculture across selected regions, focusing on representative examples to illustrate general trends, challenges, and innovations, rather than offering extensive data coverage of the mentioned countries and subregions for clarity. Our purpose is to provide a comparative overview of OA across regions with well-established systems, such as the EU and the USA, and emerging systems, including India and Malaysia.

## Organic agriculture in India, Malaysia, Europe, the USA

### India

**Figure 2:**
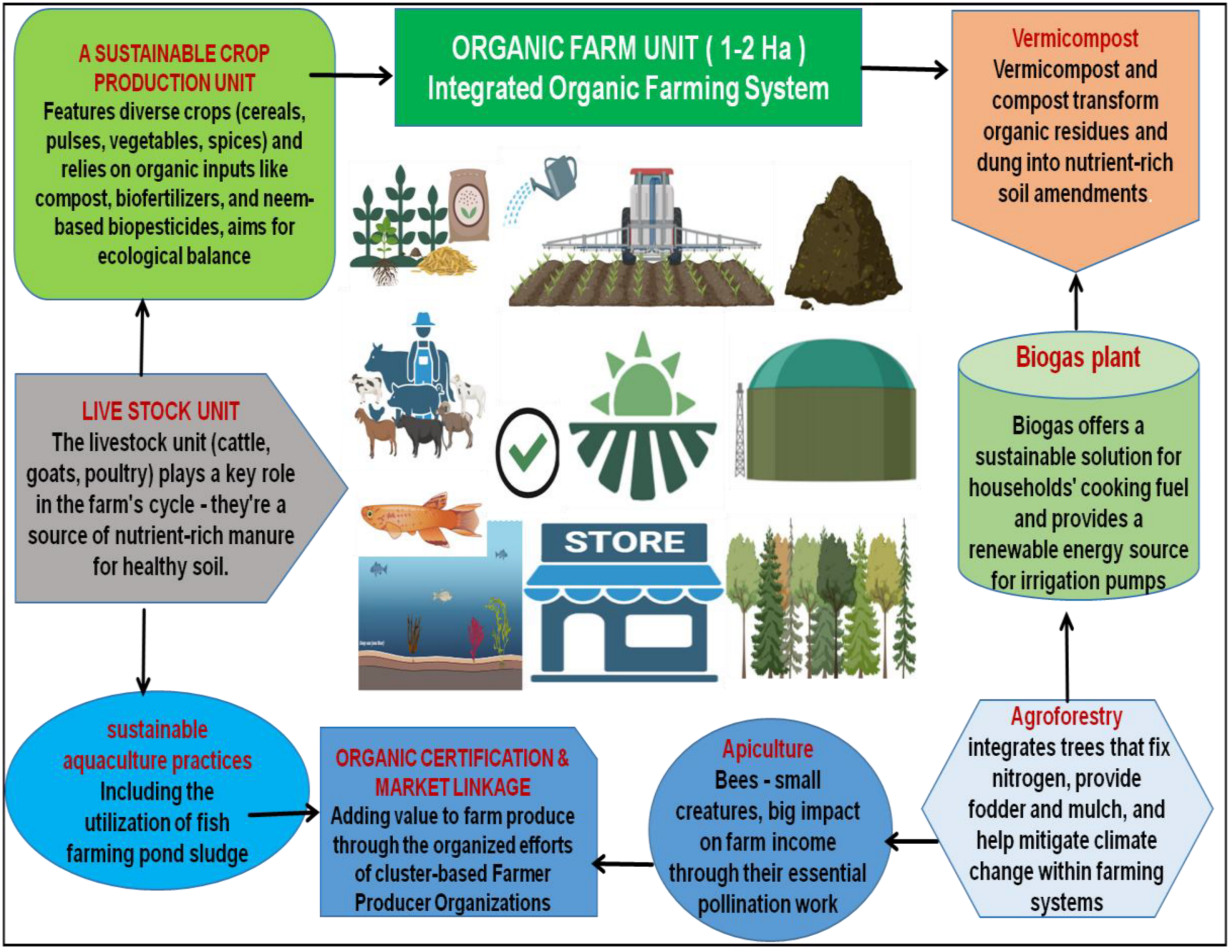
Flowchart illustrating an integrated organic farming system across one to two hectares. Key components include a sustainable crop production unit using organic inputs; a livestock unit providing manure; vermicomposting transforming waste into soil amendments; a biogas plant providing renewable energy; agroforestry integrating nitrogen-fixing trees; apiculture for pollination; and sustainable aquaculture practices using fishpond sludge. Organic certification and market linkages add value through farmer organizations. The system emphasizes ecological balance and sustainability Krishnaprabu, 2019; Devi et al., 2024; Tay et al., 2024).

India’s success is rooted in its rich agro-biodiversity and traditional knowledge systems. However, the country faces challenges such as high certification costs and limited availability of untreated seeds, as well as risks of contamination from genetically modified crops (Sivaraj et al., 2017). Despite these challenges, several successful organic agriculture models have emerged, each employing diverse strategies (**Figure 2**). For example, the **“**Integrated Organic Farming Systems (IOFS)” model combines crop-livestock integration and crop rotation to optimize resource use and enhance soil health (Krishnaprabu, 2019; Devi et al., 2024). Another model, “Zero Budget Natural Farming (ZBNF),” is a cost-effective approach that utilizes local resources like manure and biofertilizers (Duddigan et al., 2022). Additionally, community-supported agriculture (CSA) is gaining traction in urban and semi-urban areas, establishing direct connections between producers and consumers (Tay et al., 2024).

Recent studies propose strategic models aimed at empowering Indian farmers through the adoption of organic agriculture. For instance, a survey of “Socioeconomic Models” found that farmers in Tamil Nadu reported improved livelihood security after adopting organic practices. Key factors contributing to this success include educational background, media exposure, and extension support (Philip and Sivaraj, 2018). Within this framework, the Participatory Guarantee Systems (PGS) model serves as a cost-effective alternative to formal certification, allowing smallholders to access organic markets (Devi et al., 2024). Furthermore, the concept of Market-Led Organic Agriculture emphasizes direct procurement and value addition, thereby promoting farmer profitability (Naik et al., 2024). India holds the record for having the highest number of farmers (producers) engaged in organic farming, among 187 countries. India accounts for 30% of the global total of organic producers, covering 2.30 million hectares. The total area under organic cultivation stands at 2,759,660 hectares, with 1,160,650 farmers practicing under the PGS system and 1,599,010 under India Organic certification, along with 1,703 processors and 745 traders (NCONF, 2025).

### Malaysia

Malaysia’s organic agriculture sector has made significant strides by implementing innovative strategies, including urban agriculture and integrated pest management (IPM). Malaysia’s interest in organic practices gained momentum in the 1990s, with formal recognition in policy emerging in the early 2000s (Somasundram et al., 2016). Initially, the focus was on key crops such as rice and palm oil (Vasudevon, 2013; Parveez et al., 2020). Over time, projects in peri-urban and urban agriculture, including the GK Organic Farm, have played a crucial role in promoting polyculture and agroforestry systems. GK Organic Farm, located in Malaysia, is one of the region’s most prominent certified organic farms, renowned for its integration of sustainable soil management, organic composting, and community-supported agriculture practices. These projects integrate vegetable, herb, and aquaculture farming with eco-tourism initiatives (Keyvanfar et al., 2020).The growth of organic agriculture in Malaysia has also been supported by certification processes under the Malaysian Organic Scheme (MOS) and financial incentives provided through national agricultural plans. As a result, the area of organic land increased from 250 hectares in 2005 to over 1,200 hectares by 2010 (Tiraieyari et al., 2014). Case studies on Malaysian organic practices further illustrate these developments. Organic farms identify many integrated practices that are both sustainable and productive. For instance, GK Organic Farm employs polyculture practices, intercropping sweet potatoes and water spinach to enhance biodiversity and promote organic pest control (Yeng, 2016). Composting and vermicomposting are practiced extensively, converting farm waste into nutrient-dense compost and reducing reliance on external inputs (Ramaloo et al., 2018; Keyvanfar et al., 2020). Non-chemical approaches are also prioritized in pest and disease control, such as the use of natural predators and biopesticides derived from neem and other native plants (Srinivasan, 2012). Community participation is also essential, with programs such as the Bandar Harapan Enterprise providing training in organic agriculture to marginalized groups and promoting sustainable sources of income (Aini et al., 2005).

More recently, the Malaysian Agricultural Research and Development Institute (MARDI) has developed and implemented integrated organic farming models that enhance system resilience through nutrient cycling and biodiversity-based innovations. These models incorporate animal husbandry, specifically the rearing of goats and chickens within the farming system, to support closed-loop nutrient management. Animal excreta are composted with farm waste to generate on-site nutrient inputs, reducing dependency on external fertilizers and contributing to farm sustainability and self-reliance.

MARDI’s model has been implemented in both lowland and highland agroecosystems. In lowland settings, the model integrates six key components: (1) crop production as the main economic driver, (2) ruminants such as cattle and goats, (3) high-density farming, (4) pollination services through stingless bees (*Trigona* spp.), (5) zero waste management practices, and (6) alternative income sources such as mushroom cultivation. The model is underpinned by agroecological principles, including the use of attractive and repellent plant species to regulate pest populations, which is referred to as agricultural eco-engineering.

At highland locations, such as the Cameron Highlands, the model emphasizes the agricultural eco-engineering concept of planting. Beneficial plant species are established at crop borders and between rows to serve as companion crops. This strategic planting not only enhances ecological interactions and supports pest regulation but also reduces the need for organic and biological pesticides, thereby lowering production costs while maintaining yield stability. Together, these approaches represent a comprehensive agroecological strategy for sustainable organic farming in Malaysia’s diverse environments.

### Europe

**Figure 3:**
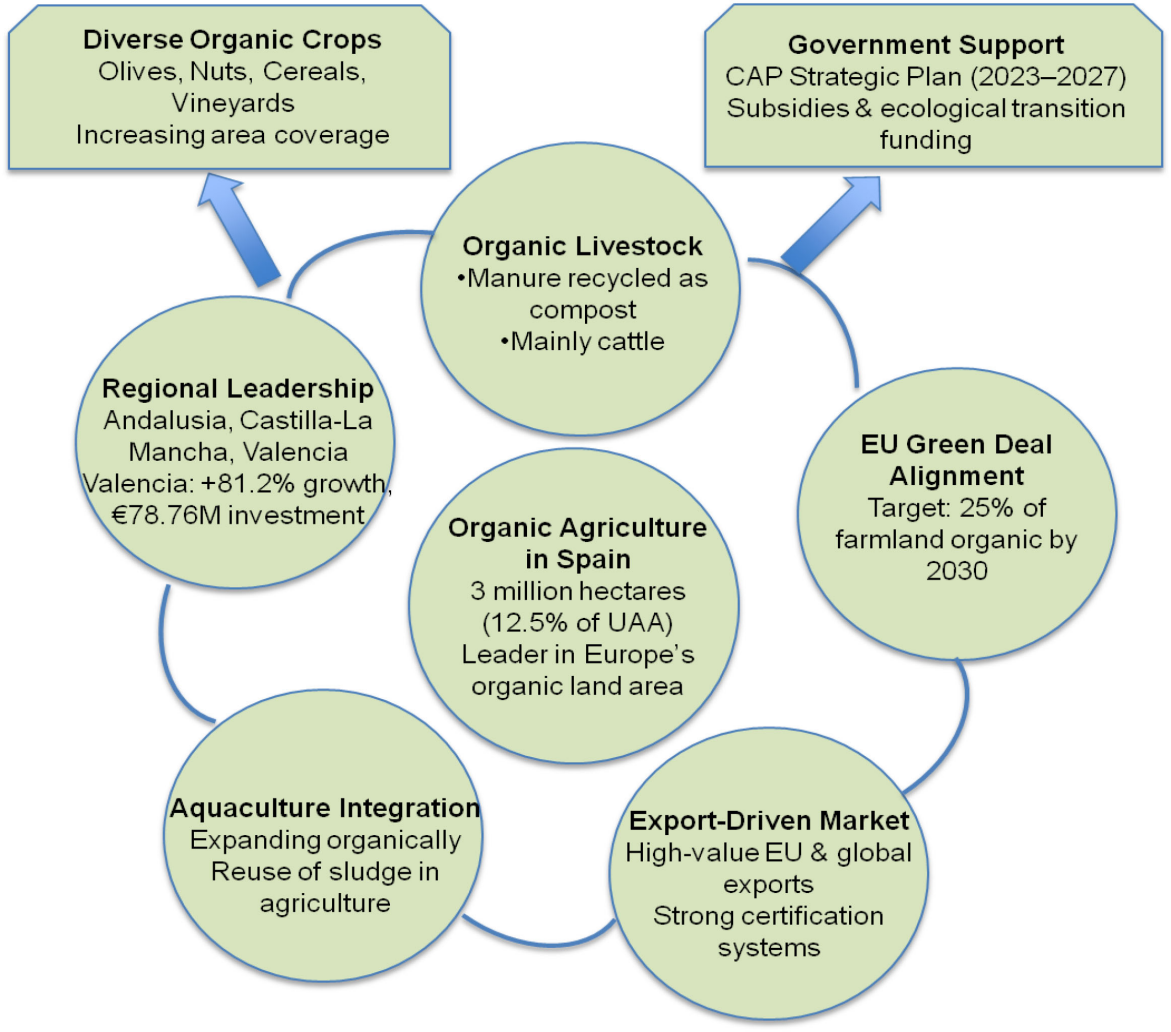
Diagram showing factors influencing organic agriculture in Spain. Central circle highlights 3 million hectares dedicated to organic farming. Surrounding factors include diverse organic crops, government support, EU Green Deal alignment, organic livestock, regional leadership, aquaculture integration, and export-driven market. Key statistics and initiatives are mentioned for each factor.

The European Union (EU) has positioned itself as a global leader in organic agriculture through various policy-oriented strategies, including the European Green Deal and the Farm to Fork Strategy (F2F) (**Figure 3**). These initiatives aim to transition the EU towards climate neutrality and sustainable food systems by 2050 (Purnhagen et al., 2021; Schebesta, 2023). One of the primary objectives of these strategies is to increase the share of organic agriculture to 25% of total utilized agricultural land (UAA) by 2030. However, as of 2021, organic agriculture represented only 10% of UAA, with notable disparities among member states (Krajewski et al., 2024).

In 2025, Spain regained its position as the leader in organic agriculture within the European Union, surpassing France, which had held the top spot since 2020. By 2023, Spain had achieved 2,991,881 hectares of organic land, representing an 11.8% increase from the previous year, according to the FiBL/IFOAM report. France ranked second with 2.77 million hectares, followed by Italy in third place. On a global scale, Spain is now ranked sixth in terms of total organic area, following Australia, India, Argentina, Uruguay, and China (Willer et al., 2025). From its inception, Spanish organic production has been focused on export, primarily driven by high consumer demand from Central Europe. It is estimated that approximately 80% of organic produce is exported, mainly to EU countries (89.2%), with the largest markets being Germany, France, and the UK (Bisquert and Meira, 2019; Weiss, 2013; FAO, 2025).

Spain has one of the most advanced organic agriculture models in Europe, marked by significant growth and substantial state support. As of 2023, over 3 million hectares, approximately 12.5% of Spain’s arable land, are dedicated to organic agriculture, with Andalusia having the most significant area under cultivation. This model encompasses a diverse range of crops, including olive trees, nuts, cereals, and vineyards, as well as organic livestock and emerging aquaculture practices (**Figure 3**). Fueled by the EU’s Green Deal and Spain’s CAP Strategic Plan for 2023–2027, the country aims to increase its organic land coverage to 25% by 2030. Regional initiatives, such as Valencia’s Ecological Production Plan, further promote the adoption of sustainable and certified organic production, positioning Spain as a leader in this field (Castillo-Díaz et al., 2024).

The Organic Agriculture Law is governed by the EU Organic Agriculture Regulation (EC) No. 834/2007 (European Commission, 2022a). This regulation prohibits the use of synthetic pesticides, chemical fertilizers, and genetically modified organisms (GMOs). It also promotes biodiversity, soil conservation, and higher animal welfare standards (Krajewski et al., 2024). Economic assistance plays a crucial role, with the EU providing approximately 3 billion euros annually through the Common Agricultural Policy (CAP) and rural development programs. This support averages around 200 euros per hectare for certified organic fields (European Commission, 2022b). Additionally, Short Food Supply Chains (SFSCs) have emerged as a prominent model, particularly in countries like Italy, Spain, and Greece. These local production methods enhance economic resilience and reduce the environmental footprint (Krajewski et al., 2024).

The adoption of organic agriculture varies across the EU, with Austria, Estonia, and Sweden leading the way, each surpassing 20% of their Utilized Agricultural Area (UAA) devoted to organic farming (**Table 1**). In contrast, Italy, Czechia, and Latvia are expected to see steady growth and may reach over 20% by 2030. Meanwhile, Malta and Ireland are currently lagging, with organic share percentages of less than 5% due to limited agricultural land, high population density, and insufficient policy prioritization (Krajewski et al., 2024).The Netherlands has a robust organic agriculture sector, characterized by substantial research output, and ranks among the leading EU countries in terms of organic research and production area (Eurostat, 2024; FiBL statistics, 2023). Despite advancements in organic farming, several challenges persist. One significant issue is the yield gap, as organic yields typically achieve only about 80% of those in conventional systems, particularly for high-input crops such as wheat and potatoes (De Ponti et al., 2012). Economic constraints also impact farmers during the transition to organic practices, leading to income uncertainty (Seufert and Ramankutty, 2017). Sound policy enforcement remains essential, necessitating increased economic incentives, targeted plans for regions with underperformance, and consumer education programs to stimulate market demand (Purnhagen et al., 2021). While organic production in the EU is expected to grow, coverage is projected to reach only 12.1% by 2030, which is significantly lower than the anticipated 25%. Bridging this gap will require enhanced investments in research, education, and tailored policy adaptations specific to different regions (Krajewski et al., 2024).

**Table 1:** Comparison of organic certification systems India, Malaysia, Europe, and the USA.

Region	Key practices and features	Policies	Comparative highlights	References
India	IOFS, ZBNF, CSA	PGS-India, PKVY, Market-led programs	Focus on smallholders, high adoption in Tamil Nadu, and direct producer-consumer linkages.	Krishnaprabu, 2019; Duddigan et al., 2022; Tay et al., 2024
Malaysia	Polyculture, Agroforestry, Urban and peri-urban farming, IPM	MOS financial incentives	Integrated models with animal husbandry, ecotourism, and highland/lowland adaptation	Tiraieyari et al., 2014; Keyvanfar et al., 2020
Europe	Diverse crops, Short Food Supply Chains (SFSCs), livestock & aquaculture	EU Green Deal, CAP subsidies, EU Organic Regulation	Export-oriented, extensive state support, and regional disparities in adoption	Bisquert and Meira, 2019; Purnhagen et al., 2021; Krajewski et al., 2024; Castillo-Díaz et al., 2024
USA	OA, DCS, CSA	USDA NOP, federal subsidies, crop insurance	Strong domestic market, large-scale commercial practices, and high certification costs	Mpanga et al., 2021; Carlson et al., 2023; Skorbiansky (2025).

IOFS, Integrated Organic Farming System; ZBNF, Zero Budget Natural Farming; CSA, Community-Supported Agriculture; PGS, Participatory Guarantee System; PKVY, Paramparagat Krishi Vikas Yojana; MOS, Malaysia Organic Scheme; IPM, Integrated Pest Management; SFSC, Short Food Supply Chain; CAP, Common Agricultural Policy; ROA, Regenerative Organic Agriculture; DCS, Diverse Cropping System; USDA NOP, United States Department of Agriculture National Organic Program; EU, European Union.

### USA

The United States has positioned itself as a global leader in organic agriculture, with large-scale, commercial practices regulated under the USDA National Organic Program (NOP). By 2021, organic farming covered approximately 4.89 million acres (Skorbiansky, 2025). The USDA Economic Research Service reports that certified organic U.S. land increased from 1.8 million acres (728,434.16 hectares) in 2000 to 4.9 million acres (198,2959.65 hectares) in 2021 (Skorbiansky, 2025). A significant focus within organic agriculture is the organic agriculture (OA) model, which emphasizes restoring soil health and enhancing carbon sequestration (LaCanne and Lundgren, 2018). Organic farming systems have shown superior drought resilience, with yields that can exceed those of conventional agriculture by up to 100% under water-limited conditions. This is attributed to improved soil structure, better water retention, and increased mycorrhizal activity (Lotter, 2003). Additionally, diverse cropping systems (DCS), such as crop rotation, cover cropping, and intercropping, enhance biodiversity and reduce dependency on chemicals, with California leading the way in organic vegetable and fruit production (King and Hofmockel, 2017). Community-Supported Agriculture (CSA) programs have also strengthened local food systems; by 1997, over 1,000 CSAs were serving approximately 100,000 households nationwide (Paul, 2019). However, small-scale farmers face several challenges, including high certification costs, limited access to organic inputs, and insufficient extension support, as many agents lack training in organic methods (Larson and Duram, 2000; Constance and Choi, 2010; Mpanga et al., 2021). The USDA’s NOP, established in 2000, provides a standardized regulatory framework that mandates annual farm inspections, enforces the National List of Allowed and Prohibited Substances, and offers support programs such as subsidies and crop insurance for farmers transitioning to organic practices (Lotter, 2003). Despite these initiatives, ongoing concerns persist regarding the accessibility of certification for smallholders and the exclusion of certain agricultural practices from organic standards, underscoring the need for increased federal funding for research and outreach programs (Mpanga et al., 2021) (**Table 1**).

## Comparative policy synthesis

Regionally, policy structures influence the adoption of organic agriculture through unique mechanisms. In Europe, policy-led incentives, most notably the Common Agricultural Policy (CAP) subsidies, which range from $218 to $490 USD per hectare, are the main drivers of growth, compensating farmers for the transition and ecosystem services they provide. The United States also relies on market incentives, such as consumer price premiums, private investment in certification, and federal crop insurance programs administered by the USDA NOP. The OA focus has resulted in robust retail expansion. Still, it has also caused imbalances between large-scale commercial farms and smallholders. In India, it is socially and institutionally led, supported by initiatives such as PKVY and PGS, which reduce certification entry barriers for smallholders and reinforce producer-consumer linkages. Malaysia is an example of a hybrid model, with public sector incentive-based measures (e.g., myOrganic certification) complemented by private partnerships and eco-tourism-driven marketing approaches. Collectively, these models identify that. In contrast, market-driven mechanisms, such as US state-driven subsidies, like those in the EU, will promote mass adoption but rely on budget sustainability. In contrast, market-driven mechanisms, such as those in the US, ensure commercial viability but risk excluding small producers. Mixed incentive systems, as seen in India and Malaysia, can potentially offer more inclusive and context-sensitive channels for scaling organic agriculture.

## Organic certification systems

**Figure 4:**
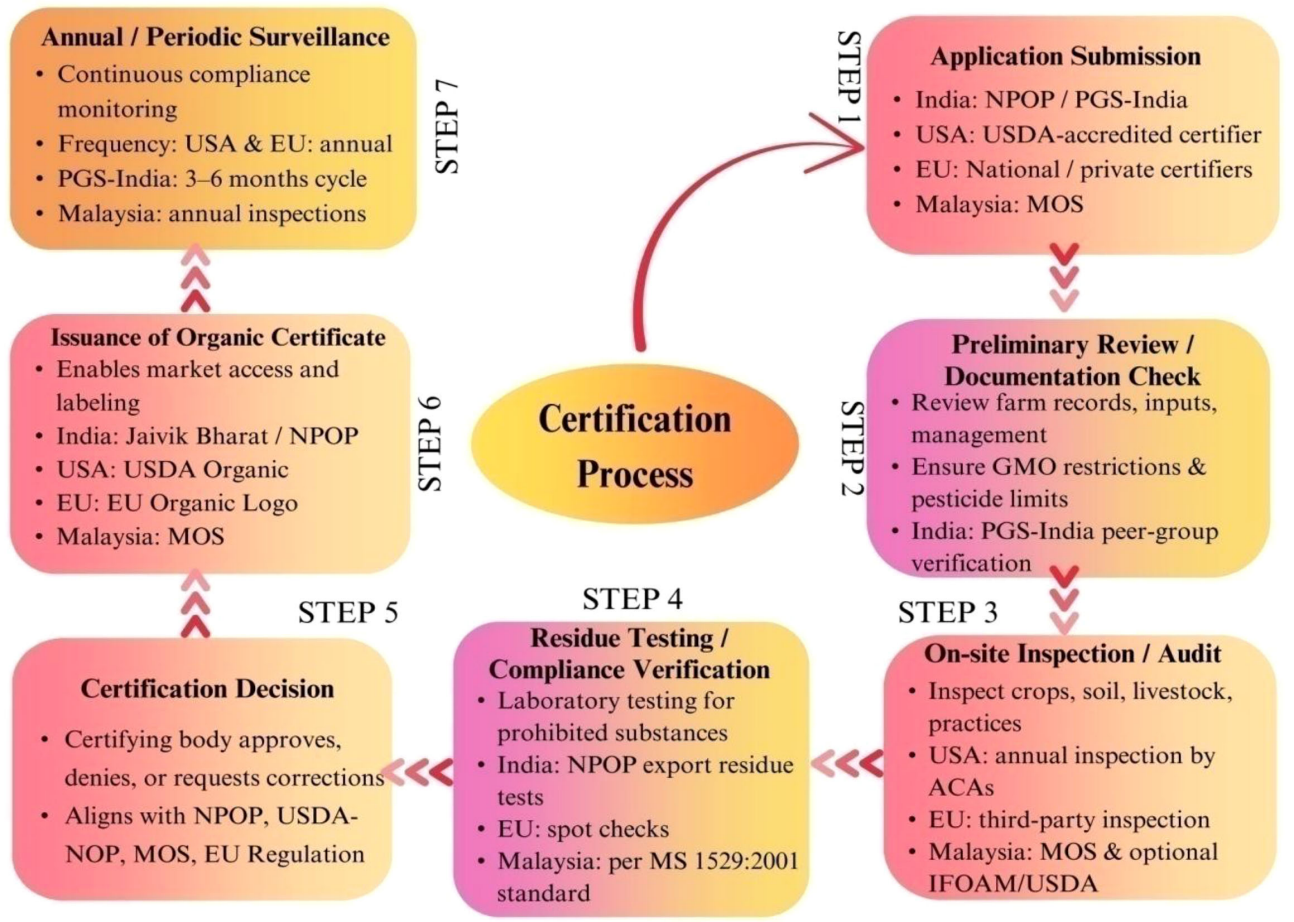
Flowchart illustrating the organic certification process in seven steps: Application Submission, Preliminary Review, On-site Inspection, Residue Testing, Certification Decision, Issuance of Organic Certificate, and Annual Surveillance. Each step details specific actions and geographical requirements for India, USA, EU, and Malaysia. The chart highlights the continuous process of compliance from application to monitoring.

Organic certification is a vital part of the organic agriculture movement, ensuring that agricultural products adhere to established standards of environmental stewardship, health, and ethical production. Certification processes can differ by region, with systems such as the USDA NOP in the United States and the EU Organic Regulation governing organic food production and trade (**Figure 4**) (Skrodzka, 2017; Brito et al., 2022). Various frameworks in the United States, Malaysia, India, and Europe are summarized below (**Table 2**).

**Table 2:** Comparative analysis of organic certification systems in Malaysia, India, the USA, and Europe.

Feature	Malaysia	India (NPOP, PGS-India)	USA (USDA NOP)	Europe (EU organic regulation)	Reference
Certification authority	DoA, IFOAM, private certifiers	APEDA-accredited certifiers, PGS-India	USDA-accredited certifiers	National agencies and private certifiers	Rahmat et al. (2021); Arifin et al. (2024)
Regulation criteria	MOS, IFOAM, USDA-NOP, traceability, soil, and input records	NPOP and PGS-India; residue limits, crop inspection	USDA-NOP, input audits, label compliance	EU Organic Reg., traceability, input control, environmental health	USDA-AMS (2023); IFOAM (2024); USDA-India (2024); NCONF (2025).
Certification time(months)	12–24	12–18	12–36	12-24	USDA-AMS (2023); Malaysian Organic Certification Scheme (myOrganic) (2024).
Certification cost (USD) (annually)	~$630	~$300–$600	$700–$3,000	$1,300–1,400	USDA-AMS (2023); NCONF (2025); Arifin et al. (2024).
Market focus	60% imported organics	Primarily export-oriented	Strong domestic market presence	High domestic demand and exports	Aziz et al., 2021; Khoh (2012); Pekala (2020)
Subsidies for farmers	Limited financial support	Partial financial support (PKVY scheme)	Limited federal support	~$214–$482 per hectare under CAP	Rahmat et al. (2021)
GMO policy	Strictly prohibited	Strictly prohibited	Strictly prohibited	Strictly prohibited	Mohamed Haris et al. (2018)

DoA, Department of Agriculture; IFOAM, International Federation of Organic Agriculture Movements; MOS, Malaysia Organic Scheme; NPOP, National Program for Organic Production; PGS, Participatory Guarantee System; USDA NOP, United States Department of Agriculture National Organic Program; EU Organic Reg., European Union Organic Regulation; CAP, Common Agricultural Policy.

The differences in certification systems across regions are summarized in **Table 2**. In India, fragmented supply chains and low domestic demand hinder adoption (PGS-India/NPOP). Compliance challenges also differ for smallholding farmers in India; they face high costs and limited market access (IFOAM - Organics International). In the USA, USDA NOP-certified farms encounter extensive record-keeping, strict auditing, and label compliance issues, which can be burdensome for small and medium-scale operations (Simonsen and Lillywhite, 2014; Mpanga et al., 2021; National Organic Coalition, 2023). The certification process also requires documentation of all farm activities, including sources of inputs, crop histories, pest management records, and sales logs, which must be retained for a minimum of five years for audit purposes. Such regulatory compliance augments administrative expense and time spent, especially for smallholders (USDA-AMS, 2023). However, these requirements became necessary following a major fraud case in which conventional grain was sold as organic, resulting in a $140 million scandal (U.S. State Attorney's Office, Northern District of Iowa, 2019). In response, the USDA’s Agricultural Marketing Service made strict rules on traceability, record audits, and supply-chain verification to prevent policy violations (USDA-AMS, 2023). In Malaysia, compliance with MOS standards, alongside international certifications such as USDA-NOP or IFOAM, presents challenges in maintaining consistent record-keeping and meeting multiple regulatory requirements simultaneously (Somasundram et al., 2016). European farmers must navigate both EU-wide organic regulations and additional national private standards, leading to higher administrative costs and potential discrepancies across member states (Barrett et al., 2001). These requirements also entail large-scale record-keeping of inputs and certification documents, contributing to compliance costs for small-scale producers (European Commission, 2022a; 2022b; FiBL, 2023a; 2023b).

### Organic certification framework in the USA

The USDA National Organic Program (NOP), established in 2002, regulates organic production, handling, and labeling in the United States through third-party certification and rigorous oversight (USDA-AMS, 2023; USDA, 2025a; 2025b). NOP regulations specifically prohibit the use of synthetic pesticides, genetically modified organisms (GMOs), ionizing radiation, and sewage sludge in organic production (USDA-AMS, 2023). For livestock, the requirements include providing organic feed, ensuring access to the outdoors, and avoiding the use of antibiotics or growth hormones (Skrodzka, 2017). For propagation stock, the USDA-NOP requires certified organic seed and planting stock. However, suppose no equivalent organic variety is available on the market. In that case, untreated conventional seed may be used if it is free from substances and coatings prohibited by organic production (USDA-AMS, 2023). The certification process involves collaboration with USDA-approved certifying agents (ACAs), an annual inspection, and the maintenance of records for a minimum of five years (Brito et al., 2022).

The U.S. uses three main certification mechanisms for organic farming: (1) Third-Party Certification (TPC), which is the most extensive system where independent certifiers verify compliance with USDA organic standards (Hatanaka et al., 2005). (2) Internal Control Systems (ICS), which enable group certification for smallholder farmers to reduce costs (Meinshausen et al., 2019), and (3) Participatory Guarantee Systems (PGS), a decentralized, community-based model that relies on trust and peer verification (Roggio and Evans, 2022).

While the certification system is effective, it faces several challenges. One major issue is the cost, as certification fees can range from $700 to $3,000 annually, creating a financial burden that limits the participation of small and medium-sized farms in the organic market (Brito et al., 2022; National Organic Coalition, 2023; Skrodzka, 2017). The complexity of documentation is another challenge, as farmers must keep detailed records of inputs, soil amendments, and pest control measures (Brito et al., 2022). Additionally, consumer confusion over labels such as “organic,” “natural,” and “non-GMO” undermines trust. In contrast, the growing presence of large corporate organic farms that practice industrial organic methods raises concerns about the dilution of organic values (Anastasiou et al., 2019; Grasseni, 2025). This confusion is further exacerbated by the development of equivalent marketing terms, such as “sustainable” and “regenerative,” which are not covered under the USDA organic regulations but are widely used interchangeably in the retail marketplace. Additionally, only items labeled as “100% Organic” or “Organic” (containing at least 95% organic ingredients) can bear the USDA Organic Seal. In comparison, those carrying the label “Made with organic ingredients” (containing ≥70% organic ingredients) and those with certain organic elements (<70%) are not permitted to use the seal, creating further confusion for shoppers (USDA-AMS, 2023).

### Organic certification framework in India

Indian organic certification is a crucial vehicle for regulating production, ensuring market access, fostering consumer trust, and promoting sustainable agricultural practices (Aulakh and Ravisankar, 2017; Sharma and Gautam, 2025). The National Program for Organic Production (NPOP) and the Participatory Guarantee System (PGS) regulate it, aligning India’s certification system with global standards and prioritizing the needs of smallholder farmers. NPOP, established in 2001 and administered by the Agricultural and Processed Food Products Export Development Authority (APEDA), mandates third-party certification for organic exports to destinations such as the EU, USA, and Japan, with more than 28 accredited certifying bodies operating in India (Verma et al., 2022). Approved by global institutions such as IFOAM, the EU Organic Regulation, and the USDA-NOP, NPOP certification enables Indian organic commodities to be exported to international markets. It encompasses a wide range of farming areas, including crops, livestock, wild harvest, processing, and organic aquaculture (Gupta et al., 2020; NPOP, 2025). Yet, with high certification costs of between Indian Rs 25,000 and Rs 50,000 (annually, together with rigorous residue testing standards), small-scale farmers are financially constrained from participating (Sailaja and Manohari, 2021).

To address these issues, the Indian government introduced the Participatory Guarantee System (PGS-India) under the Paramparagat Krishi Vikas Yojana (PKVY) in 2015. This system serves as a cost-effective, community-based alternative to third-party certification (Katto-Andrighetto et al., 2019). Unlike the NPOP which relies on external auditors, PGS-India employs a farmer-group certification approach. In this model, farmers certify one another’s adherence to common organic principles. Additionally, PGS-India features an accelerated certification process that usually takes 3 to 6 months to complete, compared to the 2 to 3 years required by NPOP (Hill, 2016). However, PGS-India faces challenges, such as low market visibility, as leading retailers and institutional buyers often prefer third-party certification. Furthermore, the adoption of PGS-India varies by state, resulting in inconsistencies in enforcement (Hill, 2016; Sailaja and Manohari, 2021).

In addition to government-led certification programs, various state-level and private initiatives also impact India’s organic certification scheme. Jaivik Bharat, launched in 2017 by the Food Safety and Standards Authority of India (FSSAI), serves as an umbrella brand that aims to standardize organic labeling (Mishra et al., 2020). Private organizations such as EcoCert and INDOCERT provide certification services for both domestic and export markets of organic products (Kaur and Shah, 2018; Sherief et al., 2021). Furthermore, state-driven initiatives, such as the Sikkim Organic Mission, have taken significant steps in this area, with Sikkim becoming India’s first fully organic state. It has established a regulatory system to support organic farmers (Humphrey, 2024).

### Organic certification framework in Europe

The EU’s organic certification system is regulated by harmonized rules across all member countries, ensuring consistency in organic production, processing, and labeling (Brzezina et al., 2017). EU Regulation 2018/848 outlines the framework for this certification, prohibiting the use of synthetic pesticides, fertilizers, and genetically modified organisms (GMOs) in organic agriculture. This regulation also requires the use of certified organic seeds and planting material whenever it is available; only if an equivalent organic seed is not commercially available may untreated conventional seeds be utilized under temporary derogation. Yet, several EU member states, such as Austria, Denmark, and France, have increasingly limited or phased out this derogation by creating national organic seed databases and supply-chain enforcement systems (Solfanelli et al., 2021; European Commission, 2022a; 2022b; Research Institute of Organic Agriculture [FiBL], 2023).

It also mandates third-party certification from recognized agencies within each member state (Migliorini and Wezel, 2017). Animal welfare standards are a key component of the certification process. Organic livestock must have access to pasture, experience limited use of antibiotics, and organic feed (Migliorini and Wezel, 2017). Products that contain at least 95% organic content are required to display the EU organic logo, which helps ensure consumer transparency and differentiate these products in the market (Willler et al., 2024). Additionally, the Common Agricultural Policy (CAP) plays a crucial role in supporting organic certification by providing financial incentives to promote organic farming.

Organic subsidies ranging from €200 to €450 per hectare, depending on the country and crop type, help offset the costs associated with certification and production challenges (Fusco, 2021). The Rural Development Program (RDP) offers supplementary financial assistance to aid conventional farmers in transitioning to organic practices. Furthermore, the Common Agricultural Policy (CAP) integrates organic agriculture into broader agroecological programs, linking certification incentives to policies that promote climate resilience and biodiversity conservation (Brzezina et al., 2017). However, despite these initiatives, variations in CAP payments across regions result in uneven adoption of organic certification in Europe. Some member states offer greater financial incentives than others, leading to unequal development of organic agriculture (Murphy et al., 2022).

In addition to the EU-wide certification, several member states have implemented more stringent private and national organic labels, adding another layer of differentiation to the organic market. For instance, Germany offers schemes such as Bioland, Naturland, and Demeter, which impose stricter requirements for biodiversity and soil fertility beyond EU standards. These labels appeal to consumers seeking stronger environmental and ethical assurances (Sahm et al., 2013). Similarly, France’s Agriculture Biologique (AB) label has sustainability requirements that exceed EU legislation, ensuring compliance with higher organic standards (Schader et al., 2012). Scandinavian countries also have national organic certification schemes, including Denmark’s Ø-label and Sweden’s KRAV certification, which mandate higher environmental sustainability practices (Pekala, 2020). While these private and national labels enhance customer trust and promote higher organic agricultural standards, they can also lead to market segmentation and increase compliance costs for farmers who seek multiple certifications to access different markets (Murphy et al., 2022).

To strengthen the EU organic certification system, it is essential to provide subsidies for small farmers, which will help lower financial barriers. Additionally, increasing domestic organic production can reduce reliance on imports. Improving fraud prevention through blockchain-based traceability would enhance transparency, while promoting biodiversity-focused organic practices instead of monocultures would ensure sustainability (Hilten et al., 2020). Moreover, raising consumer awareness and ensuring transparent labeling will further build trust in EU organic standards.

### Organic certification framework in Malaysia

The Malaysia Organic Scheme (MOS), established in 2002 by the Department of Agriculture (DoA), serves as the primary organic certification system in the country. It establishes national standards for organic agriculture, processing, and labeling, ensuring compliance with MS 1529:2001, which outlines the practices involved in organic farming (Somasundram et al., 2016). The certification process involves thorough assessments, including evaluating soil quality, implementing pest and disease management protocols, conducting residue testing, and performing farm inspections to verify adherence to organic principles (Mohamed Haris et al., 2018). Obtaining MOS certification requires third-party verification, which means that farms must maintain accurate record-keeping and traceability to ensure transparency within the organic supply chain (Rahmat et al., 2021). In addition to the MOS, farmers in Malaysia aiming for international market access can obtain certifications such as IFOAM and USDA-NOP. These certifications enable exports to the European Union and the United States (Rahmat et al., 2021). Malaysia is increasingly relying on international certification bodies, including Ecocert, to cater to diverse consumer preferences (Khoh, 2012). Furthermore, Halal-organic certification has gained importance, as it combines Islamic dietary requirements with organic food production standards, addressing the rising demand for ethically and sustainably produced Halal food (Ali and Suleiman, 2016; Ibrahim et al., 2019). These diverse certification options provide flexibility for farmers while ensuring the credibility of organic products in both domestic and international markets.

## Some successful organic agriculture models

Effective organic agriculture models depend on a combination of strong policy frameworks, market integration, and technological advancements (**Table 3**). In Europe, the organic sector is heavily influenced by policy, while the United States follows a market-driven strategy. In contrast, countries such as India and Malaysia emphasize community involvement and access to international markets.

**Table 3:** Some successful organic agriculture models in different countries in Asia, Europe and the USA.

Country/location	Key success factors		Policy support	Technology adoption	Market strategy	Metrics (yield/profit/sustainability impact)	Reference
	USA						
California	Large-scale organic farms, supply chain efficiency	USDA Organic subsidies		AI-based irrigation, robotics	Large box stores and retail chains	Organic cereal, fruit, and vegetable systems yield 10–18% less than conventional farms yet remain profitable with 5–7% price premiums. Overall, organic farms earn 22–35% higher net profits due to premium pricing.	Carlson et al. (2023) Crowder and Reganold (2015)
Vermont	Smallholder organic agriculture, CSA model	Certified by Vermont Organic Farmers (VOF), a USDA-accredited certifier; increased auditing and recordkeeping after 2019 fraud cases		Agroecology based	Direct-to-consumer sales	CSA models improve farm income stability and reduce dependence on external inputs. Economic analyses show CSA farms achieve up to 15–25% higher net income stability compared to conventional local markets.	Paul (2019); National Organic Coalition (2023); Agricultural Marketing Service (2025)
	Europe						
Denmark	Strong consumer trust, high organic food consumption		338.67 USD–510.48USD/ha subsidies, public procurement mandates	Sensor-based agriculture	Government-backed branding campaigns	In Danish long-term arable trials, involving annual cereal, legume, and forage rotations, it was found that the organic versus conventional dry-matter yield gap was approximately 30–55% compared with traditional systems across sites (Averaging Jyndevad, Foulum, and Flakkebjerg).	European Commission (2022b); Shah et al. (2017)
Bavaria, Germany	Research-led organic agriculture, export focus		EU and national subsidies	AI-based weed control, IoT irrigation	Export and domestic organic retailers	In Germany, including Bavaria, organic arable farming yields are typically 15–55% lower than those of conventional farming, depending on the crop type, soil, and management practices.	Krajewski et al. (2024); Hinzpeter (2025)
	India						
Sikkim, India	Fully organic state, policy-driven model		Free certification, government grants	Low-tech (traditional composting)	Agri-tourism and organic branding initiatives	Yield: Higher system productivity (24.6 t ha^−1^ ) Profitability: High net returns USD 4,500–4,600 ha^−1^ , Benefit–Cost ratio 1.04–1.09 Sustainability: Improved soil health -Organic Carbon 1.26%, available N 415 kg ha^−1^ , P 22.8 kg ha^−1^ , K 411.5 kg ha^−1^ ), and enhanced microbial activity compared with conventional systems,	Parikh (2023); Avasthe et al. (2020)
Andhra Pradesh, India	Low-cost agriculture model, soil health improvements		State-funded training and support	Microbial soil amendments	Local and national markets	Bio-fertilizer production reported for the year 2023–2024 is 4108 MT	Thallam (2023), (NCONF, 2025)
	Malaysia						
Cameron Highlands, Malaysia	Cool-climate farming, high-value organic vegetable exports		Free Malaysia Organic Scheme (myOrganic), certification, and support	IoT drip irrigation, hydroponics, and composting	Export-oriented Singapore	Provides insights into the differences between organic and inorganic farming, noting that organic agriculture reported lower gross margins of 13.5%, and more inputs were required for organic cultivation in Malaysia. However, organic products also reported more consumer preference and fair soil microbial diversity.	Tiraieyari et al. (2014); Rahmat et al. (2021); Yeng, 2016; Ministry of Agriculture and Food Security, Malaysia, 2024

Globally, organic farming practices vary based on policy frameworks, market dynamics, and environmental conditions. In Europe, countries such as Denmark and Germany have adopted policy-driven approaches, including subsidies and public procurement mandates, to encourage organic agriculture (EU Commission, 2022a). In the United States, states like California have developed market-driven organic sectors, while Vermont prioritizes smallholder agriculture and community-supported farming (USDA-AMS, 2023; 2025b). In India, initiatives such as Sikkim’s 100% organic state program and Andhra Pradesh’s Zero-Budget Natural Farming (ZBNF) demonstrate the effectiveness of government-led interventions and community-based approaches (Parikh, 2023; Gupta et al., 2020). Meanwhile, Malaysia has combined policy support, NGO initiatives, and private sector engagement to promote organic vegetable farming, particularly in the Cameron Highlands and peri-urban areas, where these efforts have been successful despite challenges such as land tenure and certification. Research indicates that factors such as environmental concern, education, and institutional support play significant roles in the adoption of sustainable practices among Malaysian farmers (Tiraieyari et al., 2014; Somasundram et al., 2016).

## Plant disease management in organic agriculture

Conventional agriculture harms the environment by causing soil degradation, which diminishes soil structure, fertility, and biodiversity due to the excessive use of chemical pesticides. This deterioration often increases the risk of root diseases because it reduces the population of the beneficial microbiome, necessitating interventions such as genetic resistance and soil fumigation (Van Bruggen and Finckh, 2016). In contrast, organic agriculture avoids the use of synthetic pesticides and fertilizers, thereby promoting more sustainable farming practices (Lampkin, 1990). Research shows that root diseases are generally less severe than foliar diseases in soils managed organically (**Table 4**). However, the reasons for this difference, including factors such as nitrogen supply and microbial diversity, are rarely investigated (van Bruggen et al., 2016). Disease management must be integrated with soil nutrient and environmental management. This creates both challenges and opportunities to understand the complexities of biodiversity in organic farming systems (van Bingen and Termorskuizen, 2003). Numerous detailed reviews on the management of plant diseases in organic agriculture have been published in recent years (Rawat et al., 2021; van Bruggen, 2015; van Bruggen et al., 2016; van Bruggen and Finckh, 2016).

**Table 4:** Key plant diseases in organic agriculture and their management approaches.

Disease	Pathogen	Host	Key organic management strategies	Biological controls and preventive cultural practices	References
Late blight	*Phytophthora infestans*	Potato, Tomato	Crop rotation, copper fungicides (limited use), resistant varieties, and sanitation	Application of *Trichoderma harzianum* and compost triggers Induced Systemic Resistance (ISR) in plants and suppresses pathogen growth, also, the effectiveness varies with host genotype and microbial composition	van Bruggen (2015); Mengesha (2017); Mollah and Hassan (2023)
Powdery mildew	*Blumeria graminis* f. sp. *tritici* (cereals), *Podosphaeraxanthii* and *Golovinomyces cichoracearum* (cucurbits), *Erysiphe cruciferarum* (Brassicas), *Leveillulataurica (*chili and legumes)	Cereals, cucurbits, brassicas, grain legumes	Resistant cultivars, sulfur sprays, and adequate spacing for ventilation help maintain air circulation, reducing humidity and disease spread.	Potassium bicarbonate spray, neem oil, and avoiding excess nitrogen, milk spray, and biological control using *Ampelomycesquisqualis* or *Bacillus subtilis*	Mildew (2000); Finckh et al. (2015); Mollah and Hassan (2023)
Apple scab	*Venturia inaequalis*	Apple	Resistant cultivars, sanitation, sulfur/copper sprays (restricted)	Application of *Bacillus subtilis* and *Aureobasidium pullulans* formulations	van Bruggen (2015); Köhl (2019)
Damping-off	*Pythium* spp.*, Rhizoctonia solani*	Many vegetables	Soil solarization, compost amendment, *Trichoderma*, and neem cake	Bio-priming with *Trichoderma asperellum*, proper drainage, and use of sterilized seedbeds	Rawat et al. (2021); Sharma and Gautam (2025)
Fire blight	*Erwinia amylovora*	Apple, Pear	Pruning infected branches, use of copper sprays, and resistant varieties	Sprays of *Pantoea agglomerans* or *Aureobasidium pullulans*, avoiding excessive pruning	Bastas (2015)
Take-all	*Gaeumannomyces graminis*	Wheat	Crop rotation, bio-control agents (*Pseudomonas* spp.), and reduced soil N	Incorporation of organic matter, pH management, and delayed sowing	Rawat et al. (2021); Hiddink et al. (2005)
Fusarium wilt	*Fusarium oxysporum (including* f. sp. *lycopersici*, f. sp. *cubense*, f. sp. *ciceris)*	Tomato, Banana, Chickpea, other grains, and vegetable legumes	Use of resistant cultivars, soil amendments, and crop rotation	*Trichoderma harzianum* inoculation, use of biochar, and grafting onto resistant rootstocks	Rawat et al. (2021); Bhatt et al. (2024)
Black rot	*Xanthomonas campestris*	Brassicas	Botanical pesticides, mixed cropping, and sanitation	Mixed cropping, field drainage, and pathogen-free seedlings	van Bruggen et al. (2016); Liu et al. (2022)
Downy mildew	*Pseudoperonospora cubensis* (Cucurbits), *Hyaloperonospora parasitica* (Brassicas), *Peronospora effusa* (spinach), *Bremia lactucae* (lettuce)	Cucurbits, Brassicas, spinach, lettuce	Resistant varieties, clean seed, crop rotation	Use of *Bacillus subtilis* or *Pseudomonas fluorescens*, neem oil sprays, improved ventilation, and drip irrigation to reduce humidity	van Bruggen et al. (2016); Al-Aswad and Al-Azzawi (2021)
Corky root	*Pyrenochaeta lycopersici*	Tomato	Organic amendments and crop rotation	Application of composted manure and use of suppressive soils	Zaccardelli et al. (2010); TOMI, 2024
Verticillium wilt	*Verticillium dahliae*	Tomato	Resistant cultivars, e.g., Ve-gene lines, Organic amendments, solarization, anaerobic soil disinfestation, grafting, and biocontrol	Biocontrol using *Trichoderma viride* and *Bacillus subtilis*, and crop rotation with non-hosts	Fradin et al. (2009); Testen and Miller (2018); Rahman et al. (2021)
Botrytis gray mold	*Botrytis cinerea*	Tomato, pepper, snap bean, cucurbits, and other fruits and vegetables	Responsive tomato cultivar uses of resistant or tolerant cultivars across crops, sanitation, and controlled humidity	*Trichoderma*-based biocontrol Strategies, application of compost tea, and ventilation management to reduce humidity	Luis et al. (2025)

Plant disease management in organic farming focuses on maintaining soil health and biodiversity through practices such as composting, using green manures, and crop rotation. Biocontrol agents, including *Trichoderma* and *Pseudomonas fluorescens*, effectively suppress soil-borne pathogens (Rawat et al., 2021). Successful management of apple scab using resistant cultivars and sulfur sprays illustrates how integrated organic practices can effectively control major diseases in important crops (Finckh et al., 2015). For example, Fusarium wilt in tomatoes can be managed using neem cake, farmyard manure (FYM), and *T. harzianum* (van Bruggen and Finckh, 2016). Another study highlights the importance of ecological knowledge and integrated practices in organic farming for controlling downy mildew in cucurbits. This control is achieved through the use of microbial antagonists and strict field sanitation, as organic farming (OF) promotes beneficial microbiomes in the soil (van Bruggen, 2015). Microbiome engineering offers significant benefits for organic disease management, as composting and biofertilizers can enhance plant resistance to root rot diseases by modifying rhizosphere dynamics (Akanmu et al., 2021). While conventional methods, such as neem extracts and ash, can also aid in disease management, they often face stringent certification challenges in developing countries because locally formulated biopesticides frequently lack standardized formulations, quality control, and documented efficacy data as required by organic certification agencies (Ferdous et al., 2021). Overall, holistic organic agriculture methods, such as crop rotation, field sanitation, and the careful use of copper, can effectively control diseases like late blight and fire blight when applied consistently (Rawat et al., 2021). However, it is essential to note that in some European Union (EU) jurisdictions, the use of copper in organic farming has been restricted or phased out due to environmental concerns (Tamm et al., 2022).

## Plant breeding and seed development for organic systems

Organic farmers grow food differently, so they require different seeds for their crops. In contrast to traditional cultivars bred for highly controlled environments with synthetic fertilizers, herbicides, and pesticides, organic system cultivars must withstand weeds, pests, and fluctuating nutrient availability without assistance. Perfectly bred seeds grow well without pesticides and synthetic fertilizers, as they are tailored to their specific climate and soil. Organic food should begin with organic practices; however, most organic farmers currently use seeds that are not certified organic. While certified organic seeds are mandated under the USDA National Organic Program and the EU, a provision exists for the use of untreated conventional seeds where an equal organic variety is not found. Farmers are typically requested by certifiers to have at least three commercial sources verified before such an exception is granted. The State of Organic Seed (OSA) provides a recurring report from the Organic Seed Alliance, tracking organic seed systems in the U.S. OSA publishes this progress report and action plan for expanding the organic seed supply, while developing seed grower networks and policies that work towards decentralizing ownership and power in seed systems every five years. The organic foods market is still growing rapidly, its sales reaching more than $56 billion in 2020 (a 12% expansion from 2019). This suggests that the demand for organic seeds is expected to rise soon. Yet, numerous industrial-scale organic farms continue to use untreated conventional seeds due to limited access to organic seeds and the added expense. This “conventional seed loophole” is an ongoing challenge that must be addressed through incentives to grow organic seeds, as well as policy reform.

Recent advancements in plant breeding technology have enabled the development of disease-resistant varieties suitable for organic production (Van Esse et al., 2020). For instance, Qaim (2020) demonstrates that new breeding technologies, such as marker-assisted selection, allow the incorporation of genes for resistance to fungal, bacterial, and viral diseases into susceptible cultivars. Brzozowski and Mazourek (2018) emphasize that organic systems need cultivars to address specific regional challenges. For example, cucumber lines bred for resistance to downy mildew have shown success in organic trials. Based on a genome-wide association study, we identified several tomato accessions that exhibit high resistance to Septoria leaf spot disease. Additionally, we found molecular markers linked to quantitative trait loci associated with this resistance within organic tomato production systems (Pandey et al., 2023). Similarly, Zhang et al. (2018) discuss leveraging wild relatives and landraces to enhance blast resistance in rice and improve quantitative resistance in tomatoes to achieve durable resistance. Molecular studies (Deng et al., 2020) have further revealed how resistance *R* genes and signaling networks can be utilized to develop broad-spectrum and durable resistance. Practically, resistance breeding in winter wheat has already shown economic benefits, as resistant cultivars reduce reliance on fungicides without sacrificing yield over multiple seasons (Lüttringhaus et al., 2021).

The USDA-Organic Agriculture Research and Extension Initiative (OREI) has had a positive impact on U.S. agriculture by funding projects that enhance the production of organic crops and livestock (https://www.nifa.usda.gov/grants/, National Institute of Food and Agriculture, 2025). OREI encourages innovation in areas such as soil management, pest and disease management, and animal nutrition while improving access to organic markets (https://www.nifa.usda.gov/grants/). Key impacts of OREI include enhanced profitability and productivity, as well as innovation and diversification. The initiative provides region-specific solutions to address unique challenges, such as soil acidity in the South and pest pressure in the Northeast. Additionally, OREI promotes improved sustainability practices by fostering research that supports soil health, biodiversity, water conservation, and pollution reduction. Some examples of research findings from OREI-funded projects are outlined below.

The **TOMI 3** project, launched in September 2024 and funded by the USDA, aims to help organic tomato growers manage foliar diseases while enhancing fruit quality and protecting the environment (https://eorganic.info/node/12999). It focuses on improving biocontrol agents, establishing regional breeding programs for desirable traits, developing decision-support tools for disease management, and providing training for farmers and Extension educators. The TOMI1 and TOM2 projects were completed. This initiative builds on ten years of research to address issues such as inconsistent biocontrol effectiveness and the need for more suitable tomato varieties for organic conditions (https://eorganic.info/node/12999). Tomato plants respond differently to the beneficial fungus *Trichoderma harzianum*in relation to the foliar pathogen Botrytis cinerea, depending on their genotype. This study compared the responsive *Solanum pimpinellifolium* LA 1589 to the unresponsive *S. lycopersicum* ‘Wisconsin 55’ using RNA sequencing (Luis et al., 2025). They found that LA 1589 treated with *T. harzianum* exhibited reduced disease lesions, increased height, and greater root biomass compared to the control. Notably, upregulating specific pathways while downregulating salicylic acid was crucial for enhancing resistance to *B. cinerea*. These findings improve the understanding of *Trichoderma*’s biocontrol efficacy and provide insights into selecting genotypes with enhanced growth and resistance to pathogens (Luis et al., 2025).

Previous work by Hoagland et al. (2015), conducted under the OREI program, established a participatory organic tomato breeding project in the U.S. Midwest. This project, utilizing grower surveys and multi-year field testing, selected tomato genotypes that are flavorful and resistant to several diseases, including late blight, early blight, and Septoria leaf spot, as the primary breeding source. Open-pollinated and heirloom cultivars like ‘Arkansas Traveler’ and ‘Wisconsin 55,’ and F1 hybrids like ‘Mountain Magic’ performed well under organic management. Several breeding populations demonstrated consistent disease resistance, providing a foundation for future participatory breeding efforts with local farmers. The project highlighted the importance of direct farmer involvement and region-based selection in creating cultivars that are better adapted to organic systems (Hoagland et al., 2015; https://eorganic.info/node/12999).

To improve the productivity and market potential of organic small grains sown in both spring and fall, the research team at Pennsylvania State University, along with collaborators from the Northeast US, is conducting a study (https://portal.nifa.usda.gov/web/crisprojectpages/1032780-improving-the-productivity-resilience-and-diversity-of-organic-small-grain-production-in-the-northeast.html). This research aims to enhance the ecological and economic resilience of organic grain production systems. It is crucial to understand the relationships between integrated crop management practices and the quality of food and feed outcomes, especially in relation to fungal populations and poultry health. In another study, 36 carrot breeding lines and cultivars were evaluated over four years in two locations under organic and conventional management (Colley et al., 2025). Their results show that the management system affects carrot performance, but year-to-year variations have a greater impact than the system itself. While genetic improvements from conventional systems can benefit organic production, selection in organic systems may yield faster gains. If selection isn’t pursued in organic systems, testing cultivars under these conditions is still recommended to ensure optimal performance (Colley et al., 2025).

## Innovative technologies in organic agriculture

The need for sustainability has primarily driven developments in organic agriculture technology, climate change adaptation, and the quest for greater efficiency without relying on chemicals (**Table 5**). One of the most promising trends in this field is precision organic agriculture, which integrates artificial intelligence, remote sensing, and automated decision support tools to enhance organic farming operations (Rahmann et al., 2017; Eissa, 2024; Kaur et al., 2024). Another significant innovation is eco-functional intensification, which aims to increase farm productivity while minimizing reliance on chemical inputs. This technique emphasizes natural soil regeneration, biodiversity conservation, and more effective crop rotation, leading to more sustainable organic farming systems (Niggli et al., 2017). The use of biofertilizers, biostimulants, and pheromone-based pest control is also becoming increasingly common in organic agriculture as alternatives to synthetic fertilizers. These products, which are derived from microbial inoculants, seaweed extracts, and compost teas, have been found to improve nutrient availability, enhance plant resistance to stress, and reduce dependence on external inputs (Padel, 2001).

**Table 5:** The future of organic farming: Innovative technologies for reshaping agriculture.

Innovative technology	Description	Benefits	Reference
Eco-functional intensification	Enhancing productivity by optimizing ecological interactions rather than relying on external inputs	Increase soil fertility, biodiversity, and resilience to pests	Niggli et al. (2017)
Precision organic agriculture	Use of drones, sensors, and AI to monitor crop health and optimize input use	Reduces labor costs, enhances efficiency, and minimizes waste	Rahmann et al. (2017)
Bio-fertilizers and bio-stimulants	Replacing synthetic fertilizers with compost teas, microbial inoculants, and seaweed extracts	Enhances soil microbiome, improves nutrient availability, and reduces dependency on chemical inputs	Padel (2001)
Advanced weed management	Use of flame weeding, robotic weeding, and cover cropping to control weeds without herbicides	Reduces labor costs and maintains soil health	Karafillis and Papanagiotou (2011)
Zero till and minimum tillage organic systems	Reducing mechanical soil disturbance while integrating cover crops and mulches	Improves soil organic matter, reduces erosion, and enhances water retention	Clark (2020)
Integrative innovations	Integrating sustainable intensification, renewable energy use, and closed-loop nutrient systems	Improves productivity while reducing the environmental footprint	Rahmann et al. (2017)
Agroforestry in organic systems and technology transfer platforms	Incorporating trees and shrubs into organic cropping systems	Enhances carbon sequestration, diversifies income sources, and provides habitat for beneficial species	Niggli et al. (2017)
Smart irrigation systems	Use of AI-driven irrigation scheduling, soil moisture sensors, and rainwater harvesting	Improves water-use efficiency and reduces over-irrigation	Karafillis and Papanagiotou (2011)
Pest and disease management innovations	Use of biopesticides, pheromone traps, and natural predator augmentation instead of synthetic pesticides	Reduces pesticide residues and enhances ecosystem balance	Padel (2001)
AI-based disease detection systems	Application of machine learning and image recognition software (e.g., CNN models, smartphone apps) for early detection of plant diseases in organic fields	Facilitates quick, non-destructive, and precise diagnosis of disease, minimizing loss of yield and reducing pesticide application	Tembhurne et al. (2023); Dolatabadian et al. (2025)
Predictive analytics and decision support platforms	Application of AI and big data to predict pest/disease infestations and improve organic management planning schedules	Enhances timing of intervention measures, e.g., release of biocontrol, irrigation, etc., also increasing efficiency and sustainability	Singh et al. (2024)

## Artificial intelligence in organic agriculture

The integration of AI in organic agriculture enhances sustainability and precision through technologies such as machine learning, computer vision, robotics, and IoT sensors (Eissa, 2024; Phairoj and Tongkam, 2024; Puengsungwan, 2020). These advancements optimize pest control, soil monitoring, and irrigation, resulting in reduced input waste, improved yield forecasts, and effective responses to challenges such as labor shortages, climate variability, and soil degradation (Mansoor et al., 2025). AI-powered tools can analyze real-time data from multispectral cameras, satellite imagery, and IoT sensors to predict pest infestations before they happen (Nyakuri et al., 2025; Kaur et al., 2024). Additionally, AI-based models have decreased the risk of late-stage disease outbreaks by 40% by enabling proactive management strategies (Karunakanth et al., 2018; Phairoj and Tongkam, 2024). On the policy front, global knowledge-sharing initiatives, such as the Technology Innovation Platform of IFOAM (TIPI), have facilitated the exchange of advanced research in organic agriculture (Niggli et al., 2017; Clark, 2020). Additionally, innovations like robotic weeding, flame weeding, and the use of trap crops have been developed to manage weeds without relying on herbicides (Karafillis and Papanagiotou, 2011).In specific instances, trap crops like Slender leaf legume in the genus *Crotalaria* were utilized to control parasitic weeds, such as *Striga* spp., by promoting suicidal germination, decreasing the number of seeds present in the soil, and enhancing soil health (Mwakha et al., 2020), thus aligning this method with organic farming practices.

Several innovations, including machine learning algorithms such as convolutional neural networks (CNNs) and recurrent neural networks (RNNs), can detect early signs of plant diseases by analyzing leaf color, texture changes, and chlorophyll content (Singh et al., 2021). Robotic technologies equipped with computer vision and deep learning algorithms can identify, classify, and remove weeds without harming crops (Wang et al., 2023). Several other computer vision and AI-based technologies are being adapted for organic systems through mechanical, thermal, and robotic approaches (Duckett et al., 2018). For example, multispectral image-based detection combined with robotic actuation, such as Agri-Bot, Agri-Drone, and Agri-AVG, enables precision mechanical weeding without the use of chemicals (Shinde et al., 2024). Autonomous platforms integrating deep-learning detection with mechanical removal mechanisms are being developed and field tested (Qu and Su, 2024). Another example is a motorized tracked weeding robot for organic onion fields, which achieved high-precision crop row identification via the YOLOv8-seg model integrated with data augmentation techniques (Yang et al., 2024). Recent advances have also demonstrated AI-driven, multi-sensor frameworks that combine visual, spectral, and soil data for sustainable weed and disease management, compatible with organic standards (Bangale and Kumar, 2024; Petrovic et al., 2025).

Soil health is another critical factor in organic agriculture, and AI is increasingly utilized for real-time soil analysis and fertility predictions. AI-powered models use big data analytics to assess soil moisture levels, pH, organic matter content, and microbial diversity (Samutrak and Tongkam, 2024; Maghirang et al., 2025). Furthermore, AI plays a crucial role in optimizing the organic food supply chain by enhancing traceability, logistics, and demand forecasting. Blockchain-powered AI platforms and predictive demand models help track organic produce from farm to consumer, thereby reducing fraud (Garrone et al., 2014; van Hilten et al., 2020; Turker, 2025).

## Sustainability in organic agriculture

**Figure 5:**
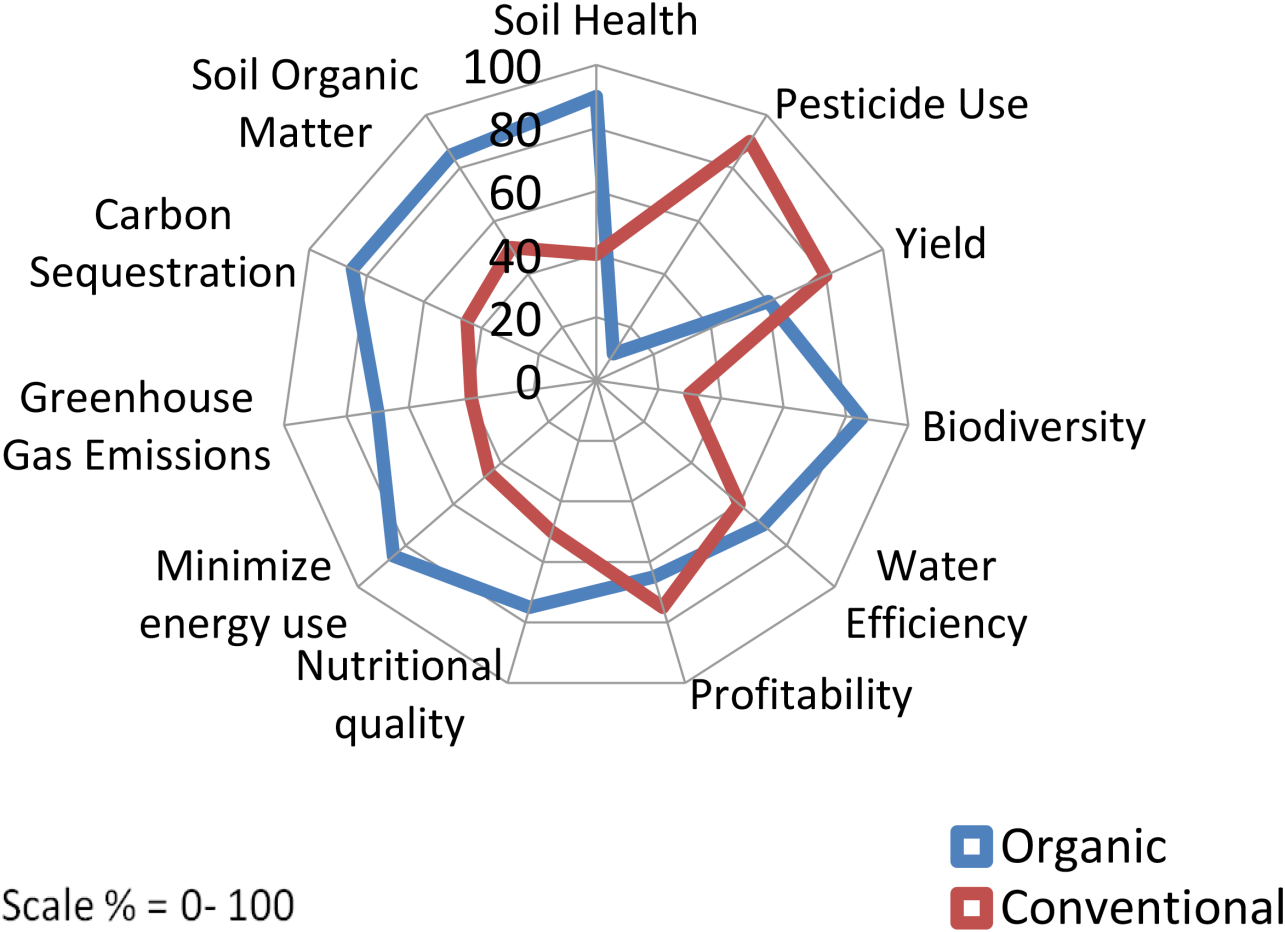
Radar chart comparing organic and conventional farming across ten criteria: soil health, pesticide use, yield, biodiversity, water efficiency, profitability, nutritional quality, minimizing energy use, greenhouse gas emissions, and carbon sequestration (Seufert et al., 2012; Reganold and Wachter, 2016; Alvarez, 2022; Boschiero et al., 2023). Organic farming scores higher in soil health, soil organic matter, carbon sequestration, and greenhouse gas emissions, whereas conventional farming excels in yield and pesticide use. Scale is from zero to one hundred.

Agriculture accounts for approximately 38% of Earth’s land and is a significant contributor to greenhouse gas emissions, biodiversity loss, agrochemical pollution, and soil erosion (**Figure 5**). Most of these issues stem from arable land, which makes up roughly 12% of the Earth’s surface (Amundson et al., 2015; Reganold and Wachter, 2016). Organic agriculture represents a sustainable approach, characterized by its balanced practices, although it inevitably involves trade-offs. In terms of productivity, organic farming typically yields about 19.2% less than conventional methods across all crops (Reganold and Wachter, 2016; Gamage et al., 2023). However, this yield gap is significantly reduced in specific circumstances, such as in drought-affected areas, low-input systems, or when cultivating certain crops like legumes and perennials (Bhullar et al., 2021). From an environmental standpoint, organic agriculture provides several benefits, including reduced greenhouse gas emissions, enhanced biodiversity, improved soil and water quality, and lower energy consumption per unit area (Parizad and Bera, 2023). Economically, organic farms often prove more profitable despite lower yields, mainly due to the price premiums they can command and reduced input costs (Gamage et al., 2023; Parizad and Bera, 2023; Robinson, 2024).To promote sustainable organic agriculture, policy tools such as subsidies, technical assistance, research funding, and educational initiatives are essential (Ume and Bahta, 2024).

Socially, organic agricultural practices contribute positively to the well-being of farmers. They help improve livelihoods, create jobs, increase wages, promote mental health, and support rural development. This impact is further confirmed by a study conducted in Europe, which analyzed data from the EUROSTAT and Eurobarometer databases to assess the effects of organic agriculture on the quality of life within the agricultural community (Stanimir, 2020).

Organic systems provide essential ecosystem services, including pollination, pest regulation, and carbon sequestration (Sandhu et al., 2010). They tend to have higher levels of soil organic matter, microbial populations, and nutrient cycling, all of which contribute to long-term agricultural resilience (Lynch, 2015). Additionally, organic farms use 15–20% less energy per unit of production compared to conventional systems. Financially, organic agriculture has significant advantages, primarily due to price premiums that are typically 29% to 32% higher than those of traditional produce (Reganold and Wachter, 2016). Organic farms are also more likely to perform better during climate extremes, such as drought, thanks to their improved soil structure and enhanced water retention (Azarbad, 2022).

Organic farming promotes environmental sustainability by minimizing the use of synthetic chemicals and reducing the need for synthetic fertilizers. Several techniques, including crop rotation, organic amendments, and integrated pest management, help restore ecosystem balance and enhance soil health (Lee et al., 2015). Meta-analyses of organic versus conventional systems in Europe and the USA indicate that organic farming typically favors greater biodiversity and superior soil health; however, in some cases, yields are lower (Tuomisto et al., 2012). From an economic perspective, organic farming has the potential for greater returns due to premium prices. Still, it also has drawbacks, including higher input costs, certification expenses, and potential yield decreases. Farmers in India and Malaysia face challenges in accessing certified organic inputs and capital, which hinder profitability (Raghavendra et al., 2025; Smith et al., 2019). Findings from Europe and the USA indicate that organic products tend to command reasonable market prices; however, the transition costs and ongoing maintenance costs of adhering to organic standards are significant deterrents (Mondelaers et al., 2009). Organic farming contributes to social adaptability by supporting community welfare, fair labor practices, and food safety (Czyżewski and Kryszak, 2022).

## Challenges in organic agriculture

Globally, organic agriculture now encompasses over 76.4 million hectares across 191 countries, with regions such as Oceania leading in land area and Europe at the forefront of policy-driven expansion (Willer et al., 2024). While organic systems offer potential environmental and economic benefits, their implementation faces several challenges, including high certification costs, yield gaps, and limitations in market access (Padel et al., 2025). OA faces several key challenges, including limited access to knowledge and markets, high certification costs, yield gaps, and policy biases favoring conventional farming (Ponisio et al., 2015; Canwat and Onakuse, 2022), though organic agriculture offers significant benefits, including reduced pollution, improved soil health, and enhanced biodiversity (Mäder et al., 2002). It promotes better nutrition for consumers and reduces farmers’ exposure to harmful chemicals. However, challenges such as lower yields compared to conventional farming, high certification costs, and market access issues hinder its widespread adoption. The varying organic standards and research gaps complicate the situation. To address these challenges, we should integrate innovative practices and technologies, such as new pest management and soil fertility strategies, to enhance yields and resilience (Reganold and Wachter, 2016). Comparative studies on the long-term impacts of organic versus conventional systems can provide valuable insights (Scialabba and Hattam, 2002). Additionally, supportive policies and effective market strategies are crucial. These steps can help maximize the potential of organic agriculture and contribute to a more sustainable food system. In India, organic certification is essential for ensuring the credibility of organic products; however, it hinders their widespread adoption. The high costs associated with accreditation disproportionately affect smallholder farmers, who face annual fees, residue testing expenses, and compliance verification issues (Panday et al., 2024). Furthermore, approximately 85% of certified organic produce is exported, resulting in low domestic demand due to limited consumer awareness (Barik, 2017). Regulatory measures further complicate the process, as bureaucratic delays in obtaining and renewing NPOP certification exacerbate the difficulties faced by farmers (Verma et al., 2022). Similarly, in Europe, certification fees vary by country and certifying body. For instance, the Soil Association (UK) charges between USD 1,200-1,500 as a base annual fee for small food businesses (Soil Association, 2025). In contrast, CERES (a Germany-based certifier) lists transaction certificate costs ranging from USD 35 to USD 100, depending on the number of product documents (CERES, 2023).

In Malaysia, organic agriculture remains limited in scale, with only 0.01% of the total agricultural land, but not due to certification costs. The Malaysian government, through the myOrganic Certification Program, offers certification completely free of charge. Additionally, farmers receive free technical support and training from the Department of Agriculture for crops (Suhaimee et al., 2016; DOA, 2025), the Department of Veterinary Services for animal husbandry practices in livestock farms, and the Department of Fisheries for aquaculture, covering fish and other aquatic organisms. Despite growing consumer interest, over 60% of organic products in Malaysia are imported due to limited domestic production capacity and stringent certification barriers (Rahmat et al., 2021). These factors diminish the competitiveness of local farmers. Additionally, consumer confusion regarding organic labels complicates market growth, as many people mistakenly believe certified organic products are labeled as “natural” or “chemical-free” (Rahmat et al., 2021). In contrast, while organic agriculture in the United States often follows a commercial, large-scale model, small and mid-sized farms face challenges related to high certification costs and lower initial yields during the transition phase (Mpanga et al., 2021). Climate risks also pose significant challenges, particularly in regions that rely on monoculture systems (Brito et al., 2022). To address these barriers, policy reforms are necessary to streamline certification processes, provide financial assistance to smallholders, and enhance domestic market awareness to support sustainable organic agriculture.

Challenges such as a lack of farmer training, consumer unawareness, and variable regulatory standards can impede social gains. Research in Malaysia emphasizes the need for capacity development and building trust among surrounding communities to facilitate the more robust adoption of sustainable practices (Tiraieyari et al., 2014). Limited market access to certified organic outlets and high competition from non-organic products pose challenges for market expansion in India, Europe, and the USA (Pandey and Singh, 2012; Peng, 2019). Organic inputs, such as compost, biofertilizers, and certified seeds, are more costly, making them unaffordable for small-scale farmers, especially in Malaysia and India. On the other hand, preserving certification integrity and transparency is crucial to establishing consumer trust, particularly in the European and US markets, where misleading labeling can make products appear less trustworthy (Thallam, 2023).

## Conclusion

Organic agriculture offers a sustainable and eco-friendly alternative to conventional farming, which often relies on chemical inputs such as pesticides and fertilizers. Various models of OA practices adopted worldwide demonstrated their adaptability and effectiveness. In India, initiatives such as the Paramparagat Krishi Vikas Yojana (PKVY) and Zero Budget Natural Farming (ZBNF) support small farmers through participatory approval systems, including PGS. In the United States, the USDA’s National Organic Program (USDA-NOP) manages a centralized system that ensures uniform certification nationwide, thereby enhancing market confidence. European countries have taken a policy-driven approach, with the European Union establishing ambitious goals under its Farm to Fork strategy (Wesseler, 2022), aiming to convert 25% of agricultural land to organic cultivation by 2030. Meanwhile, Malaysia has formalized its certification through the Malaysia Organic Scheme, which features the myOrganic label for consumer recognition (Somasundram et al., 2016). This initiative promotes OA through consumer awareness, government incentives, and national action plans for food safety (Solaiman and Salaheen, 2019). Despite these local successes, common challenges persist, including the costs associated with certification, low technical capabilities, market limitations, and yield gaps. To address these issues, it is crucial to improve localized certification systems, invest in farmer training, and enhance market connections. Comparative evidence shows that EU subsidy-driven growth, U.S. market-led expansion, and India’s PGS low-cost model each have context-specific strengths and limitations. Hybrid frameworks combining financial incentives with market access could promote global adoption (Castillo-Díaz et al., 2024). As international awareness of environmental and health concerns increases, coordinated policy support, consumer activism, and robust infrastructure will be vital for scaling up organic agriculture. Strengthening policy institutions that cover smallholder farmers’ certification, market connections, and organic input subsidies will be critical in increasing adoption. More intensive international cooperation among researchers, policymakers, and farmers will be crucial to align organic standards and drive global adoption of sustainable agricultural practices. To attain these objectives, future research should be focused on 1) studies related to the assessment of AI and precision organic agriculture technologies, 2) low-cost microbial biocontrol formulation development, and 3) breeding for climate-resilient varieties. Some of the policy recommendations include harmonizing international certification standards, subsidizing smallholders through government-backed programs, and implementing digital tracking tools to enhance label transparency. Similarly, global collaboration, primarily through initiatives such as IFOAM’s Technology Innovation Platform (TIPI) and organic partnerships led by the FAO, will play a crucial role in sharing innovations, fostering research collaboration, and building a resilient global organic agriculture network.
